# Delayed Presentation of Intussusception with Perforation after Splenectomy in Patient with Blunt Abdominal Trauma

**DOI:** 10.1155/2013/510701

**Published:** 2013-12-17

**Authors:** Ibrahim Afifi, Hassan Al-Thani, Sajid Attique, Sherwan Khoschnau, Ayman El-Menyar, Rifat Latifi

**Affiliations:** ^1^Department of Surgery, Section of Trauma Surgery, Hamad General Hospital, P.O. Box 3050, Doha, Qatar; ^2^Clinical Research, Section of Trauma Surgery, Hamad General Hospital, P.O. Box 3050, Doha, Qatar; ^3^Clinical Medicine, Weill Cornell Medical School, P.O. Box 24144, Doha, Qatar; ^4^Department of Surgery, Arizona University, Tucson, AZ 245005, USA

## Abstract

Adult intussusception (AI) following blunt abdominal trauma (BAT) is a rare surgical condition. We present a case of delayed diagnosis of ileocecal junction intussusception with a perforation of small bowel in a 34-year-old male with a history of fall from height. Initial exploratory laparotomy revealed shattered spleen requiring splenectomy. Initial abdominal computerized tomography scanning (CT) scan showed dilated small bowel with no organic obstruction. Patient started to improve with partial distention and was shifted to rehabilitation unit. On the next day, he experienced severe abdominal distention and vomiting. Abdominal CT showed characteristic intussusception at the distal ileum. Secondary exploratory laparotomy revealed severe adhesions of stomach and small bowel to the anterior abdominal wall with dilated small bowel loops and intussusception near the ileocecal junction with perforation of small bowel. The affected area was resected and side-to-side stapled anastomosis was performed. Though small bowel intussusception is a rare event, BAT patients with delayed symptoms of bowel obstruction should be carefully evaluated for missed intussusception.

## 1. Introduction

Intussusception is the telescoping of one segment of the gastrointestinal tract into an adjacent one and usually occurs in children [1]. John Hunter was the first to describe the clinical and pathological characteristics of intussusceptions. Sir Fredrick Treves proposed the first management plan for intussusception which is being practiced to date [2]. Unlike children in whom most cases are idiopathic, the majority (80%) of adult intussusception (AI) cases have an underlying cause which could be due to development of tumors, fibrosis after surgery, and Meckel's diverticula. Cases following blunt abdominal trauma are rare [3]. AI is relatively uncommon with different clinical presentation, diagnosis, and management compared to childhood intussusception [4]. AI is often diagnosed late during emergency laparotomy and the delayed diagnosis may be attributed to nonspecific presentation such as chronic colicky pain and intermittent partial intestinal obstruction associated with nausea and vomiting [5]. AI mainly needs surgical treatment which includes bowel resection with prior reduction of intussusception [4].

The increased utility of computerized tomography scanning (CT) helps in early evaluation of patients with abdominal pain and confirms the diagnosis for the possible AI without subsequent delay [1]. Definitive diagnosis of intussusception is possible due to its marked diagnostic appearance on CT imaging [6]. It appears as a complex soft-tissue mass, consisting of the outer intussuscipiens and the central intussusceptum. Though intussusception has a particular appearance on CT, it is difficult to identify the true etiological factors associated with its development [7]. Also, a high index of suspicion is required for diagnosing adult intussusception with nonspecific abdominal symptoms. However, such patients with history of prior episodes of partial intestinal obstruction should undergo CT examination to rule out intussusception [6].

In the literature, small bowel intussusception due to blunt abdominal trauma (BAT) has not been extensively reported and only few case reports are described [8]. Herein, we present a case of ileocecal junction intussusception with perforation of small bowel following BAT in an adult male.

## 2. Case Report

A 34-year-old male patient presented with BAT with history of fall from 10-meter height. On presentation, the patient was hypotensive (blood pressure of BP 81/43 mmHg) with heart rate of 130 beat per min. He had depressed fracture of left frontoparietal skull bone, contusion with abrasion of left side of face, mild chest wall hematoma, and abdominal guarding. Patient was intubated immediately with rapid sequence induction. Focused abdominal sonography for trauma (FAST) was positive; chest X-ray showed left subcutaneous emphysema with fractures of the left 6th, 7th, and 10th ribs. Chest tube was inserted and massive transfusion protocol was activated. Initial laboratory investigations showed an INR of 1.2, low pH 7.16, base excess −9.4, low hematocrit (17%), high myoglobin (2549 ng/mL), creatinine 139 umol/L, low hemoglobin (10.1 g/dL), high white blood cell count (25.9 × 10^3^ u/L), normal platelet (246 × 10^3^ u/L), and high sensitive troponin T (90 ng/L). Patient was shifted to operation theatre immediately.

An exploratory laparotomy was performed which revealed shattered spleen requiring splenectomy; otherwise, formal exploration showed no other findings.

Postoperative laboratory investigation showed hemoglobin of 11.5 g/dL, WBC 13000 u/L, platelet 192000 u/L, creatinine 64 umol/L, myoglobin 1324 ng/L, and high sensitive troponin T 189 ng/L. Follow-up laboratory findings after 6 hours revealed low hemoglobin (8.5 g/dL), normal WBC (7300 u/L), and platelet count (130000 u/L).

A CT scanning of the head showed comminuted depressed fracture of left frontoparietal bone with minimal subdural hematoma, subarachnoid hemorrhage, tiny hemorrhagic contusions of left temporal area, and fracture of left zygomatic arch. Neck and chest CT scanning revealed evidence of C6 facet fracture and fractures of left 5–8 ribs anteriorly and left 6-7, 9–12 ribs posteriorly with left hemo- and pneumothorax and bilateral lung contusions. With a smooth course in trauma intensive care unit (TICU), the patient was started enteral feeding through nasojejunal feeding tube after 24 hours and then he was extubated 2 days later. After removal of nasojejunal tube, the patient developed abdominal distention; for that nasogastric tube was inserted and 1100 mL fluid was removed initially. After a couple of days in the surgical ward, the patient showed a wax and wane course of abdominal distention. All serum electrolytes were within normal range.

**Figure 1:**
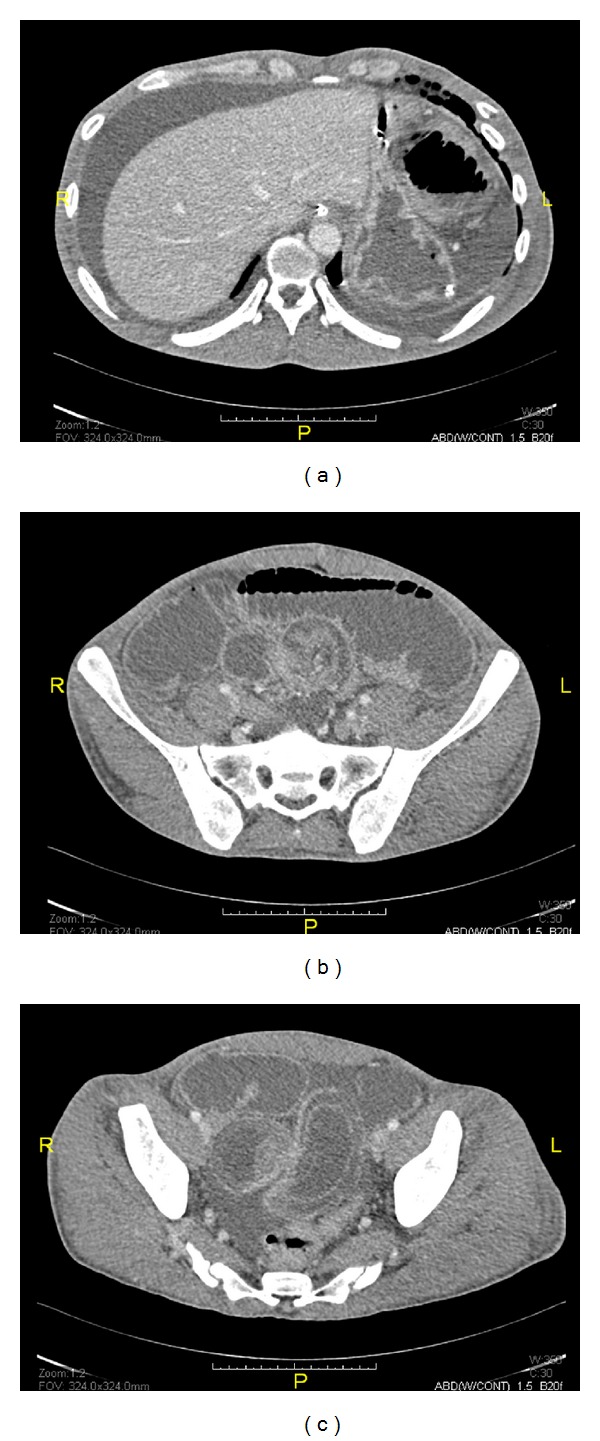
Abdominal CT scanning on day 9 of admission (day 5 after symptoms) showing peritoneal effusion, severely dilated small bowel, and suspected intussusception of the distal Ilium.

**Figure 2:**
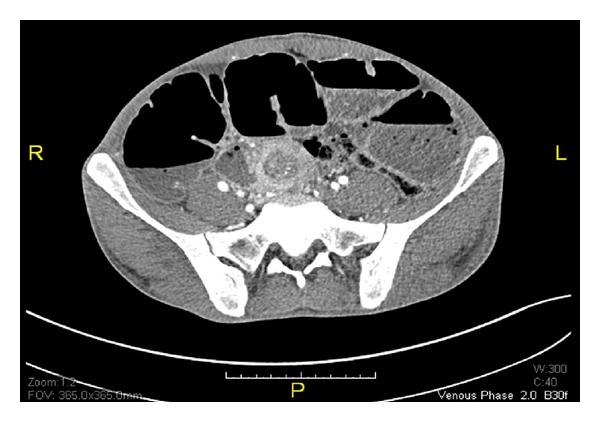
Abdominal CT scanning on day 19 (day 15 after symptoms) showing severely dilated small bowel and characteristic intussusception features: “target lesion” or “doughnut sign” and sausage-shaped mass.

**Figure 3:**
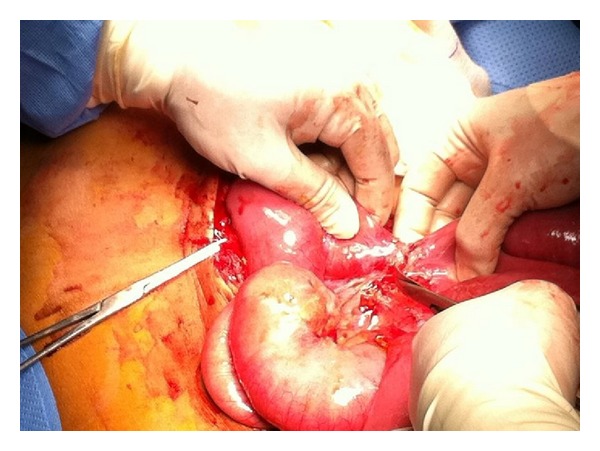
Intraoperative findings showed intussusception at ileocecal junction with perforation of small bowel.

Further CT scan of the abdomen showed dilated small bowel with no organic obstruction (Figure 1). Finally, he started to improve with partial distention and the nasogastric tube was removed. A clear fluid was commenced and gradually advanced to full diet owing to the patient tolerance. Two days later, he experienced severe abdominal distention and vomiting, and therefore he was returned back to TICU and another abdominal CT scan with trial of oral contrast was performed and showed characteristic intussusception at the distal ileum (Figure 2). Patient declined operative intervention and so he was kept on nasogastric decompressing tube and antibiotics. Later on, the patient agreed on exploratory laparotomy that revealed severe adhesions of stomach and small bowel to anterior abdominal wall with dilated small bowel loops up to 9 cm with collapse 1 meter from the ileocecal junction. There was an intussusception of 1.5 cm length at 1 meter from the ileocecal junction with perforation of small bowel proximal to it (Figure 3). The affected area was isolated and almost 3 L of fecal material was evacuated. This area was resected and side-to-side anastomosis was fashioned using gastrointestinal anastomosis (GIA) linear cutting staplers (sized 100 mm). Oversewing of the anastomotic staple line with 3-0 Vicryl sutures was done. Omental defect was closed with 3-0 Vicryl sutures. Peritoneal cavity was irrigated with 5 L of warm saline and then the abdomen was closed. Histopathological study of small bowel specimen (9 × 3 cm) showed foci of transmural necrosis and inflammation with ulceration. The postoperative course of the patient was unremarkable and he was discharged home in a stable condition.

## 3. Discussion

Small bowel intussusception in BAT patients is uncommon and few cases were published. Historically, Leichtenstern reported a series of 326 cases of intussusception; of these cases, only 8% had past history of trauma as an etiology for intussusception [9]. The mechanism of traumatic intussusception is not well defined yet. Komadina and Smrkolj [10] described that the generation of localized bowel spasm following abdominal trauma, abnormal peristalsis, and intramural hematoma might be the possible mechanism for the development of small bowel intussusception. Trauma causes adrenergic stimulation of the sympathetic nerves of the gastrointestinal tract with a resultant spasm of the sphincters and segmental spasm of bowel. The direct blunt abdominal trauma causes positive wave pressure which gives rise to enough increased peritoneal cavity pressure to drive many spastic areas into adjacent areas of relative dilatation. Other factors that can be added to the development of intussusception include recently ingested meal, severe fright, and the possible existence of autonomic nerve imbalance in cases of severe stress of trauma. Yet, it is apparent that without trauma as the trigger mechanism, these factors never would have produced remarkable lesions.

Duncan and colleagues [11] identified 6 cases of intussusception as a cause of intestinal obstruction after laparotomy among abdominal trauma patients. Five cases were diagnosed with jejunojejunal intussusception; one had jejunoileal and one had ileocolic intussusception. Manual reduction was successfully used to treat all patients and recurrence was not observed in any of cases [11]. Bashir and Lynch [12] reported a case of double jejunojejunal intussusception causing intestinal obstruction after blunt abdominal trauma. The patient recovered completely with operative manual reduction. Berne et al. [13] reported a case of double intussusception after laparotomy for penetrating liver injury. The authors suggested that intussusception can be reduced manually without the need for surgical resection.

A recent case report described an ileoileal intussusception after BAT in patient presented with symptoms of intestinal obstruction after a fall from a ladder [14]. Though the authors resected the necrotic bowel segment, they have advocated the manual reduction to overcome the possible complications of surgical resection.

Benjelloun et al. [3] presented another case of ileoileal intussusception with Meckel's diverticulum. Meckel's diverticulum was a lead point for intussusception in BAT patients, and trauma with disturbed bowel motility was the unlatching mechanism of intussusception [3]. The authors performed a diverticulectomy with small bowel resection to attain successful outcome. Another BAT case presented after motor vehicle crash and showed jejunal intussusception on abdominal exploration. The patient was managed by manual reduction with unremarkable postoperative course [8].

Controversial findings have been reported for the exact treatment of adult intussusception. However, surgical resection has been advocated by many investigators [15–17]. However, Kitamura et al. [18] suggested simple reduction for posttraumatic and idiopathic intussusceptions where no obvious pathological cause is suspected in the bowel [4]. In our case, surgical resection was used to manage the intussusception at ileocecal junction. The affected area was resected using staplers and side-to-side anastomosis.

In conclusion, the delayed presentation of small bowl intussusception after BAT is uncommon phenomenon. The diagnosis is difficult due to nonspecific and episodic symptoms which require a high index of suspicion. Abdominal CT is the modality of choice for diagnosis. Treatment requires resection of the small bowel if it is nonviable. Therefore, BAT patients with symptoms of bowel obstruction should be carefully evaluated for the possibility of intussusception.
